# Substance Use and Adherence to HIV Preexposure Prophylaxis for Men Who Have Sex with Men^1^

**DOI:** 10.3201/eid2412.180400

**Published:** 2018-12

**Authors:** Martin Hoenigl, Sonia Jain, David Moore, Deborah Collins, Xiaoying Sun, Peter L. Anderson, Katya Corado, Jill S. Blumenthal, Eric S. Daar, Joel Milam, Michael P. Dubé, Sheldon Morris, for the California Collaborative Treatment Group 595 Team

**Affiliations:** Medical University of Graz, Graz, Austria (M. Hoenigl);; University of California, San Diego, California, USA (M. Hoenigl, S. Jain, D. Moore, X. Sun, J.S. Blumenthal, S. Morris);; Department of Health and Human Services, Long Beach, California, USA (D. Collins);; University of Anschutz Medical Campus, Aurora, Colorado, USA (P.L. Anderson);; Harbor UCLA Medical Center, Torrance, California, USA (K. Corado, E.S. Daar);; University of Southern California Keck School of Medicine, Los Angeles, California, USA (J. Milam, M.P. Dubé)

**Keywords:** substance use, adherence to HIV preexposure prophylaxis, PrEP, HIV, viruses, HIV and other retroviruses, sexually transmitted infections, methamphetamine, alcohol, injection drug use, dried blood spots, adherence, men who have sex with men, MSM, transgender women, persons who injected drugs

## Abstract

The effectiveness of oral HIV preexposure prophylaxis (PrEP) strongly depends on maintaining adherence. We investigated the association between substance use and PrEP adherence, as well as incident sexually transmitted infections (STIs) in a high-risk cohort of 394 participants (391 men who have sex with men and 3 transgender women) who were enrolled in a PrEP demonstration project. We assessed baseline and ongoing substance use over a 48-week period for stimulants and nonstimulant substances and for each substance separately. We measured PrEP adherence by using dried blood spots to obtain levels of tenofovir diphosphate. No differences in these levels were found between substance users and nonsubstance users. Baseline stimulant use was strongly associated (odds ratio 3.4; p<0.001) with incident STIs during the study. Thus, PrEP adherence was not decreased by substance use. Because substance users had increased rates of STIs, indicating higher-risk behavior, they might be excellent candidates for PrEP.

## Abstract 2

Substance and alcohol use were not associated with decreased adherence.

Over the past 2 decades, substance use, in particular use of stimulants, such as methamphetamine, remains prevalent among men who have sex with men (MSM) and transgender women in the United States (*1*). Alcohol, stimulant use, and injection drug use (IDU) are independently associated with increased risk behavior and HIV acquisition among MSM and transgender women (*1*–*4*). Thus, HIV-uninfected MSM and transgender women with substance use might represent ideal candidates for preexposure prophylaxis (PrEP).

The efficacy of tenofovir disoproxil fumarate (TDF) combined with emtricitibine (FTC) for HIV PrEP has been documented in several randomized and controlled trials (*5*–*7*). In the iPrEx study, TDF/FTC reduced the risk for HIV infection in MSM by 44% vs. placebo, and a 73% lower risk of HIV infection was reported for persons who had >90% self-reported adherence (*5*), and >90% lower risk for persons who had adherence defined by tenofovir diphosphate (TFV-DP) drug levels commensurate with >4 tablets per week (*8*).

The Bangkok TDF Study randomized 2,413 persons who injected drugs (PWIDs; <5% were MSM) 1:1 to TDF or placebo, and results showed a 48% reduction in HIV seroconverison in the treatment arm (*9*,*10*). However, in that study, study participants had daily observed dosing in conjunction with substance use disorder treatment. Therefore, adherence remains uncertain among substance users without observed therapy.

The effectiveness of TDF/FTC for HIV PrEP strongly depends on maintaining adherence (*11*,*12*). Although studies have indicated that different strategies might be required for PrEP implementation for MSM who use stimulant substances and alcohol (*13*), comprehensive/demonstrative studies that evaluate adherence among MSM or transgender women using different classes of substances are lacking.

We hypothesized that, among MSM and transgender women enrolled in a randomized controlled PrEP demonstration trial, substance users would have lower levels of PrEP adherence. The objective of our study was to investigate the association between substance/alcohol use and adherence to PrEP, as well as sexually transmitted infections (STIs) and study completion, in a well-characterized high-risk cohort of MSM and transgender women who participated in the California Collaborative Treatment Group (CCTG) 595 Study.

## Materials and Methods

CCTG 595 was a randomized controlled trial of individualized text messaging versus standard care for adherence to daily TDF/FTC PrEP (http://www.clinicaltrials.gov/ct2/show/NCT01761643) (*14*). In CCTG 595, PrEP was given in combination with safety monitoring, HIV/STI testing, and risk reduction counseling. On a daily basis, participants in the intervention arm received a mixture of health promotion and factoid messages at a personally selected time consistent with when they planned to take PrEP. The study was conducted during February 2014–February 2016. Patients were enrolled at 4 medical centers in southern California (University of California San Diego, University of Southern California, Harbor–University of California Los Angeles, and Long Beach Health Department), and participants were provided with mobile phones in case they did not have a mobile phone (*14*).

Eligible participants for CCTG 595 were HIV-uninfected MSM and transgender women (age >18 years) confirmed by a negative result for an antigen/antibody assay or antibody assay plus HIV nucleic acid amplification test. Participants needed to have a persistent increased risk for HIV acquisition as determined by >1 of the following criteria: 1) >1 HIV-infected sexual partner for >4 weeks; 2) condomless anal intercourse with >3 male sex partners who were HIV positive or of unknown HIV status during the previous 3 months; or 3) condomless anal sex with >1 male partner and an STI diagnosis during the previous 3 months. Participants were required to have acceptable laboratory test values during the previous 30 days; exclusion criteria included active hepatitis B. Study visits occurred at baseline and at weeks 4, 12, 24, 36, and 48 for the primary outcome. Study participants were allowed to continue receiving the study drug past week 48 until the last participant completed his or her week 48 visit.

At each visit, we collected data by using a confidential in-person interview and computer assisted survey self-report (e.g., Patient Health Questionnaire 9 [PHQ9]). We found no significant differences in the primary adherence outcome between the 2 study arms (72.0% in text messaging arm vs. 69.2% in standard of care; p = 0.58), in adequate adherence at week 12 (91.7% vs. 85.6%; p = 0.07) or week 48 (83.4% vs. 81.6%; p = 0.77), or in baseline substance use (p = 0.11) or Drug Abuse Screening Test (DAST10) result (p = 0.30) (*14*).

For this analysis, we included randomized CCTG 595 participants who had completed the baseline substance use questionnaire (n = 394) to examine associations with substance use over 48 weeks and used dried blood spot (DBS) intracellular TFV-DP levels as a biologic measure of PrEP adherence (*15*). We assessed substance use during the previous 3 months at baseline and week 4, 12, 24, 36 and 48 visits by using a Substance Use Screening Questionnaire (SCID). Each substance variable was categorized into no use, some use (1–4 times), and frequent use (>5 times) on the basis of the frequency of use during the previous 3 months. We also analyzed use of combined stimulant substances (i.e., poppers, methamphetamine, cocaine, ecstasy, amphetamine, and other stimulants); nonstimulant substances (i.e., heroin, other opioids [e.g., hydrocodone bitartrate/acetaminophen and oxycontin], sedatives, antianxiety drugs, hallucinogens, dissociative drugs, and inhalants); and any substances (i.e., stimulant and nonstimulant substances listed previously, not including alcohol and marijuana use).

We assessed problematic use at baseline by using the DAST10 and the Alcohol Use Disorders Identification Test (AUDIT). DAST10 score was grouped into 3 categories: no or low problems, DAST10 score <3; moderate problems, DAST10 score >3–<6; and substantial or severe problems, DAST10 score >6). AUDIT score was grouped into 3 categories: <8, >8–<16, and >16. Ongoing substance use was defined as >50% of completed study visits (study had 6 regular visits) with reported use in the SCID. Ongoing substance use was defined in a hierarchical way. We first defined frequent ongoing user as reporting frequent substance use on the SCID at >50% of visits; if this criterion was not reached, we looked further at whether the study participant reported any substance use (including some and frequent) at >50% of visits; if yes, we defined them as some ongoing user; otherwise, they were counted as not an ongoing user.

We determined adherence by measuring intracellular TFV-DP levels in DBS. A TFV-DP concentration >719 fmoL/punch of a paper disk containing DBS (e.g., https://www.analytical-sales.com/DBS.html) was defined as an average of >4 tablets/week. This value is the unrounded level corresponding to 700 fmoL/punch level used in the IPREX OLE study, which reported 0 of 28 seroconversions when the TFV-DP level was >700 fmoL/punch (*16*). We determined intracellular TFV-DP concentrations at the week 12 visit and the last on-drug visit on or before the 48 week visit by using a validated method (*15*).

The primary DBS adherence outcome was a composite outcome for being adherent as defined by a DBS TFV-DP level >719 fmoL/punch (i.e., adequate adherence) at the week 12 visit and, if continued past week 12, the last study visit through week 48 (e.g., week 24, 36, or 48). Missing or not completing the visit at week 12 was considered nonadherence. If week 12 was the last study visit while receiving drug, then the adherence of the participant was based only on that 1 value. The secondary DBS near-perfect adherence composite outcome included the same composite outcome for DBS TFV-DP dose associated with taking 7 doses of TDF in the past week (>1,246 fmoL/punch) (*15*,*16*). In addition to the composite outcomes, we also performed cross-sectional analyses at weeks 12 and 48 on the basis of available samples.

As a secondary objective, we assessed whether substance or alcohol use reported at baseline impacted study completion and incident STIs during the study (i.e., measure of sexual risk behavior). STI screening assessments at baseline and every 3 months over 12 months included syphilis (serum rapid plasma reagin: and if a positive result was obtained, a confirmatory treponemal test), nucleic acid amplification test of urine, and testing of pharyngeal and rectal swab specimens for *Chlamydia* spp. and gonorrhea (Aptima; Hologic, Marlborough, MA, USA). Information about newly diagnosed STIs were communicated to participants, and referrals were made to their providers or a local STI clinic for treatment. Incident STI was defined as having positive results for gonorrhea or infection with *Chlamydia* spp. at any site or a positive rapid plasma reagin result for syphilis during the study visits after baseline.

We compared baseline characteristics, DBS adherence composites, and incident STI during the study and at study completion regarding substance/alcohol use categories by using the Fisher exact test for categorical variables and analysis of variance test. We used the Wilcoxon rank-sum test to test continuous variables. To assess the association between substance/alcohol use and outcomes (i.e., adherence, study completion, and incident), we used separate logistic regression models adjusted for study arm and other baseline factors that were associated with outcome. We assessed model discrimination by using the goodness-of-fit Hosmer–Lemeshow statistic. In addition, we used Cox regression models to study the association between baseline substance use and time to early study termination or time to first incident STI diagnosis and reported hazard ratios (HRs). We defined time to study termination as the last completed visit of a participant. Participants who did not leave the study early were censored at week 48. Participants who did not reach the event were censored at their last visit before or at week 48. A p value <0.05 was considered statistically significant. No adjustment was made for multiple comparisons. Statistical analyses were performed by using R software version 3.3.2 (http://cran.r-project.org).

## Results

**Figure 1:**
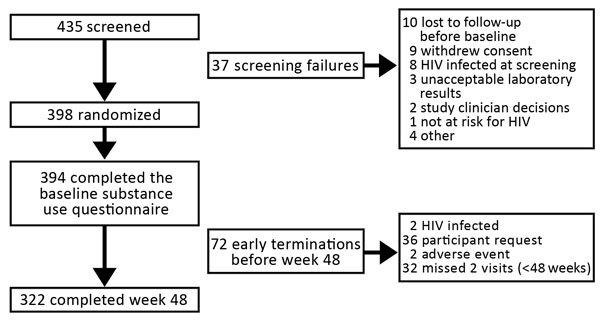
Flow chart for selection of patients from randomized controlled trial for study of substance use and adherence to HIV preexposure prophylaxis among men who have sex with men and transgender women, February 2104–February, 2016, California, USA.

A total of 394 persons participated in the study and completed their baseline substance use questionnaire (Figure 1). Of these participants, any substance use was reported by 288 (73%) and any alcohol use by 327 (83%). Overall, substance use remained relatively stable over the course of the study (e.g., 39% reported frequent substance use at baseline and 42% at week 48). Some ongoing substance use was reported by 37%, and frequent ongoing substance was reported by 38%. Participants with ongoing substance use had higher levels of depressive symptoms (PHQ9 scores) than those without ongoing substance use. We obtained demographic data and PHQ9 and DAST10 scores of subgroups with no, some, and frequent ongoing substance use (Table 1).

**Table 1:** Baseline demographic characteristics by substance use status used for assessing substance use and adherence to HIV preexposure prophylaxis among MSM and transgender women, California, USA*

Characteristic	Overall, n = 394	Substance use			p value
		None, n = 102	Some, n = 144	Frequent, n = 148	
Sex					0.191
M	391 (99)	100 (98)	143 (99)	148 (100)	
F	3 (1)	2 (2)	1 (1)	0	
Median age, y (IQR)	33 (28–41)	33 (29–40)	33 (28–41)	33.5 (28–42)	0.885
Race, n = 386†					0.238
Asian	12 (3)	2 (2)	5 (4)	5 (3)	
Black	51 (13)	20 (20)	19 (13)	12 (8)	
White	292 (76)	72 (72)	104 (73)	116 (81)	
Multiple	24 (6)	6 (6)	10 (7)	8 (6)	
Other	7 (2)	0	4 (3)	3 (2)	
Hispanic ethnicity, n = 391†	119 (30)	30 (29)	54 (38)	35 (24)	0.048
English primary language	3,786 (95)	98 (96)	133 (92)	145 (98)	0.066
Education					0.296
High school or less	35 (9)	12 (12)	15 (10)	8 (5)	
Some college	146 (37)	36 (35)	58 (40)	52 (35)	
Bachelor’s degree	132 (33)	31 (30)	42 (29)	59 (40)	
Postgraduate or advanced degree	81 (21)	23 (23)	29 (20)	29 (20)	
Household income/mo					0.434
<$2,000	84 (21)	27 (26)	31 (22)	26 (18)	
>$2,000	248 (63)	63 (62)	89 (62)	96 (65)	
Not known	62 (16)	12 (12)	24 (17)	26 (18)	
Randomization arm					0.019
Standard of care	196 (50)	60 (59)	75 (52)	61 (41)	
Text messaging	198 (50)	42 (41)	69 (48)	87 (59)	
Study site					0.660
Harbor-UCLA	48 (12)	11 (11)	15 (10)	22 (15)	
Long Beach	46 (12)	15 (15)	17 (12)	14 (9)	
UCSD	173 (44)	48 (47)	62 (43)	63 (43)	
USC	127 (32)	28 (27)	50 (35)	49 (33)	
Median PHQ9 for depression (IQR)	3 (1–7)	2 (0–5)	3.5 (1–7)	5 (2–8)	<0.001
Median baseline DAST10, (IQR)	2 (0–3)	0 (0–1)	2 (1–3)	3 (2–4)	<0.001

*Values are no. (%) unless otherwise noted. DAST, Drug Abuse Screening Test; IQR, interquartile range; MSM, men who have sex with men; PHQ, Patient Health Questionnaire; UCLA, University of California Los Angeles; UCSD, University of California San Diego; USC, University of Southern California. †Characteristics were not available for all study participants.

Overall, 89% of participants at week 12 and 83% of participants at week 48 had adequate DBS TFV-DP levels (i.e., >719 fmoL/punch); 48% of participants at week 12 and 44% of participants at week 48 had estimated near-perfect DBS TFV-DP levels (i.e., >1,246 fmoL/punch). A total of 279/394 (71%) study participants reached the primary DBS adherence composite (i.e., adequate adherence), 115/394 (29%) reached the secondary DBS adherence composite (i.e., near-perfect adherence), and 322/394 (82%) completed the study (i.e., the week 48 visit) (*14*).

Univariate analyses showed no significant difference in the primary or secondary DBS adherence outcomes between persons with or without ongoing substance/alcohol use (all p values >0.2; Table 2). There was also no significant association between baseline substance/alcohol use or between baseline DAST10 and AUDIT scores and adherence outcomes (all p values >0.5; Table 2). Similar results were confirmed in multivariable logistic regression models adjusted for study arm, race, and baseline PHQ9 scores (all p values >0.1).

**Table 2:** Associations of DAST10 and AUDIT results at baseline and ongoing substance/alcohol use with primary and secondary DBS adherence endpoints for MSM and transgender women, California, USA*

Characteristic	Primary endpoint		p value	Secondary endpoint		p value
	No	Yes		No	Yes	
Substance use baseline, n = 394						
DAST10 problems			0.80			0.55
No/low	70 (28)	179 (72)		173 (69)	76 (31)	
Moderate	37 (31)	83 (69)		86 (72)	34 (28)	
Substantial/severe	8 (32)	17 (68)		20 (80)	5 (20)	
AUDIT score			0.09			0.84
<8	81 (29)	201 (71)		197 (70)	85 (30)	
8–15	25 (26)	70 (74)		69 (73)	26 (27)	
>15	9 (53)	8 (47)		13 (76)	4 (24)	
Ongoing substance use, n = 394						
Methamphetamine			0.82			0.32
No	97 (29)	238 (71)		240 (72)	95 (28)	
Some	10 (28)	26 (72)		26 (72)	10 (28)	
Frequent	8 (35)	15 (65)		13 (57)	10 (43)	
Heroin			0.79			>0.99
No	113 (29)	275 (71)		274 (71)	114 (29)	
Some	1 (25)	3 (75)		3 (75)	1 (25)	
Frequent	1 (50)	1 (50)		2 (100)	0	
Poppers			0.54			0.66
No	62 (31)	139 (69)		146 (73)	55 (27)	
Some	32 (30)	75 (70)		75 (70)	32 (30)	
Frequent	21 (24)	65 (76)		58 (67)	28 (33)	
Cocaine			0.48			0.33
No	97 (29)	241 (71)		236 (70)	102 (30)	
Some	13 (29)	32 (71)		33 (73)	12 (27)	
Frequent	5 (45)	6 (55)		10 (91)	1 (9)	
Stimulant substances†			0.37			0.75
No	42 (30)	100 (70)		98 (69)	44 (31)	
Some	43 (33)	88 (67)		96 (73)	35 (27)	
Frequent	30 (25)	91 (75)		85 (71)	36 (29)	
Nonstimulant substances‡			0.96			0.32
No	67 (30)	158 (70)		166 (74)	59 (26)	
Some	31 (28)	79 (72)		74 (67)	36 (33)	
Frequent	17 (29)	42 (71)		39 (66)	20 (34)	
Any substance‡			0.34			>0.99
No	31 (30)	71 (70)		72 (71)	30 (29)	
Some	47 (33)	97 (67)		102 (71)	42 (29)	
Frequent	37 (25)	111 (75)		105 (71)	43 (29)	
Alcohol			0.27			0.88
No	23 (38)	38 (62)		42 (69)	19 (31)	
Some	32 (29)	79 (71)		78 (70)	33 (30)	
Frequent	60 (27)	162 (73)		159 (72)	63 (28)	

*Values are no. (%). Primary endpoint value was TFV-DP >719 fmol/punch; secondary endpoint value was TFV-DP >1,246 fmol/punch. For baseline use data for specific substances and substance classes not shown, all p values were >0.2. AUDIT, Alcohol Use Disorders Identification Test; DAST, Drug Abuse Screening Test; DBS, dried blood spot; MSM, men who have sex with men; TSF-DV, tenofovir diphosphate. †Includes poppers, methamphetamine, cocaine, ecstasy, amphetamine, and other stimulants. ‡Marijuana and alcohol excluded.

**Figure 2:**
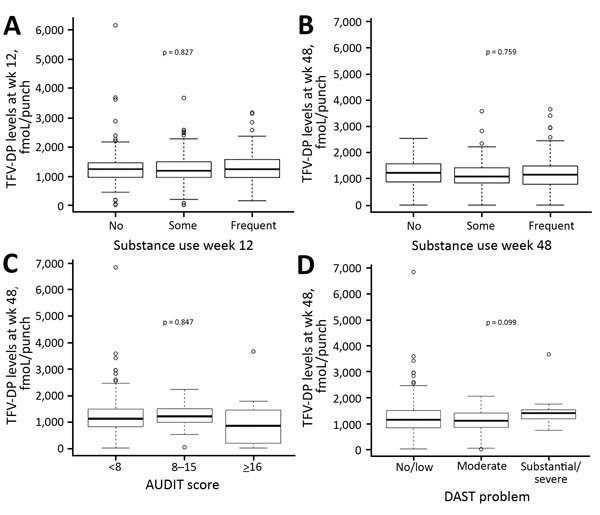
Substance use and adherence to HIV preexposure prophylaxis among transgender women and men who have sex with men, California, USA. A, B) Boxplots showing dried blood spot TFV-DP levels at weeks 12 (A) and 48 (B) for persons with no, some, and frequent ongoing substance use. C, D) Boxplots showing dried blood spot TFV-DP levels at week 48 in persons with and without alcohol (C) and substance use (D) problems, according to assessments with AUDIT (C) and DAST (D) (cross-sectional analysis). In each case, dried blood spot TFV-DP levels were compared among the 3 groups by using the analysis of variance test. Circles indicate outliers; horizontal lines within boxes indicate medians, box bottoms and tops indicate 25th and 75th quartiles; and error bars indicate levels within 1.5 times the interquartile range of the lower quartile and upper quartiles. AUDIT, Alcohol Use Disorders Identification Test; DAST, Drug Abuse Screening Test; TFV-DP, tenofovir diphosphate.

We created boxplots of DBS TFV-DP levels at weeks 12 and 48 for those with no, some, and frequent ongoing substance use (alcohol and marijuana excluded), as well as boxplots of DBS TFV-DP levels at week 48 by AUDIT and DAST10 score categories (Figure 2). We also developed cross-sectional associations of previous 3 months substance use with adequate and perfect adherence at weeks 12 and 48 (Technical Appendix Table 1). Although at week 48 persons with the highest category of AUDIT scores were significantly less likely to have adequate adherence (p = 0.03), persons who had substantial or severe substance use problems according to DAST10 were significantly more likely to reach near-perfect adherence (p = 0.04). However, when we compared DBS TFV-DP as a continuous variable between the AUDIT and DAST10 groups, differences were not significant (p = 0.847 for AUDIT and p = 0.099 for DAST10; Figure 2).

Overall 322/394 (82%) participants completed the study (Table 3). In the Cox regression model adjusting for study arm (Table 4), we found that frequent baseline substance use was significantly associated with study completion (HR for early study termination 0.541; p = 0.036) compared with persons who had no substance use; 86% of persons who had frequent substance use completed the study compared with 81% who had some substance use and 76% who had no substance use). We also calculated the same model after replacing substance use with alcohol use and methamphetamine use. Although baseline alcohol use was not a strong predictor of study completion, we found that some methamphetamine use at baseline was associated with a significantly lower likelihood of study completion compared with no methamphetamine use, but frequent methamphetamine use at baseline was not associated with study completion (HR for early study termination for some methamphetamine use 1.885; p = 0.046; 70% study completion for some methamphetamine use vs. 83% study completion for no or frequent methamphetamine use). The logistic regression models yielded similar findings (Technical Appendix Table 2).

**Table 3:** Associations of substance/alcohol use at baseline with study completion and incident STI among MSM and transgender women, California, USA*

Substance use baseline, n = 394	Study completion		p value	Incident STI		p value
	No	Yes		No	Yes	
DAST10 problems			0.59			0.043
No/low	42 (17)	207 (83)		161 (65)	88 (35)	
Moderate	25 (21)	95 (79)		63 (53)	57 (48)	
Substantial/severe	5 (20)	20 (80)		18 (72)	7 (28)	
Methamphetamine			0.15			0.037
No	56 (17)	275 (83)		211 (64)	120 (36)	
Some	12 (30)	28 (70)		17 (43)	23 (57)	
Frequent	4 (17)	19 (83)		14 (61)	9 (39)	
Heroin			0.24			0.80
No	67 (18)	310 (82)		230 (61)	115 (39)	
Some	2 (22)	7 (78)		6 (67)	1 (33)	
Frequent	3 (38)	5 (63)		6 (75)	2 (25)	
Poppers			0.25			<0.001
No	41 (21)	150 (79)		142 (74)	49 (26)	
Some	19 (17)	94 (83)		57 (50)	56 (50)	
Frequent	12 (13)	78 (87)		43 (48)	47 (52)	
Cocaine			0.31			0.18
No	58 (17)	276 (83)		211 (63)	123 (37)	
Some	9 (20)	35 (80)		24 (55)	20 (45)	
Frequent	5 (31)	11 (69)		7 (44)	9 (56)	
Stimulant substances†			0.40			<0.001
No	30 (22)	109 (78)		110 (79)	29 (21)	
Some	23 (18)	106 (82)		67 (52)	62 (48)	
Frequent	19 (15)	107 (85)		65 (52)	61 (48)	
Nonstimulant substances‡			0.95			0.33
No	42 (19)	179 (81)		140 (63)	81 (37)	
Some	19 (18)	89 (82)		60 (56)	48 (44)	
Frequent	11 (17)	54 (83)		42 (65)	23 (35)	
Any substance			0.18			<0.001
No	25 (24)	81 (76)		81 (76)	25 (24)	
Some	25 (19)	110 (81)		76 (56)	59 (44)	
Frequent	22 (14)	131 (86)		85 (56)	68 (44)	
Alcohol			0.13			0.28
No	16 (24)	51 (76)		47 (70)	20 (30)	
Some	21 (22)	74 (78)		57 (60)	38 (40)	
Frequent	35 (15)	197 (85)		138 (59)	94 (41)	


*Values are no. (%). DAST, Drug Abuse Screening Test; DBS, dried blood spot; MSM, men who have sex with men; STI, sexually transmitted infection. †Includes poppers, methamphetamine, cocaine, ecstasy, amphetamine, and other stimulants. ‡Marijuana and alcohol excluded.

**Table 4:** Cox regression models used for assessing substance use and early study termination and incident STIs among MSM and transgender women, California, USA*

Model	HR (95% CI)	p value
Model 1		
Intervention arm (receiving individualized texting for adherence to daily TDF/FTC)	1.377 (0.862–2.200)	0.180
Baseline some substance use (any)	0.743 (0.426–1.293)	0.293
Baseline frequent substance use (any)	0.541 (0.304–0.961)	0.036
Model 2		
Intervention arm	0.924 (0.671–1.272)	0.626
Age	0.973 (0.955–0.992)	0.005
Baseline some stimulant use	2.690 (1.727–4.190)	<0.001
Baseline frequent stimulant use	2.604 (1.665–4.072)	<0.001
Positive STI test result at baseline	1.450 (1.031–2.039)	0.033

*Model 1 assessed the effect of baseline substance use on early study termination. Model 2 assessed the association of stimulant use and incident STIs during the study. FTC, emtricitibine; HR, hazard ratio; MSM, men who have sex with men; STI, sexually transmitted infection; TDF, tenofovir disoproxil fumarate.

In an explorative analysis, we focused on 39 (9.9%) persons who left the study early (before the week 24 visit). We found that persons who had some baseline methamphetamine use had a greater tendency to leave the study early (17.5% left the study early vs. 4.3% who had frequent methamphetamine use and 9.3% who had no methamphetamine use), but frequent baseline substance use tended to be associated with a lower tendency to leave the study early (5.2%; all p>0.05). 

Overall, 152 (39%) of 394 participants were given a diagnosis of an incident STI during the study. By using univariate analysis (Table 3), we found that incident STIs occurred more frequently in participants with some and frequent stimulant use at baseline (incident STIs occurred in 48% of both groups vs. 21% in persons with no stimulant use at baseline; p<0.001). This difference was driven by use of poppers (52% incident STIs in persons with frequent popper use and 50% in persons with some popper use vs. 26% in persons with no popper use; p<0.001). We also found significantly higher rates of incident STIs in those with some methamphetamine use (58% STI incidence vs. 36% in persons with no methamphetamine use and 39% in persons with frequent methamphetamine use; p = 0.037). No difference was found for alcohol use.

By using Cox regression models adjusting for study arm, age, and baseline STI status, we found that stimulant use was strongly associated with incident STI during the study (HR 2.7 for some use, 2.6 for frequent use; both p<0.001) (Table 4). We also obtained significant results when stimulant use was replaced with popper use (HR 2.3 for some use, 2.5 for frequent use; both p<0.001) or any substance use (HR 2.1 for some use, p = 0.002; HR 2.0 for frequent use, p = 0.004). When we replaced stimulant use with methamphetamine use, some methamphetamine use was a significant predictor of incident STI (HR 1.9, p = 0.005), but frequent use was not a significant predictor. In contrast, alcohol use was not a strong predictor of incident STI. Logistic regression models yielded similar findings (Technical Appendix Table 2).

## Discussion

We investigated the association between substance/alcohol use and adherence to PrEP, as well as study completion and incident STIs, in a high-risk cohort of mostly MSM who participated in a randomized controlled PrEP adherence trial. Three main findings are evident. First, substance use was not associated with decreased adherence to PrEP, as measured by TFV-DP in DBS. Second, baseline frequent substance use was associated with higher likelihood of study completion. Third, baseline stimulant use was strongly associated with higher rates of incident STIs during the study, suggesting greater sexual risk behavior in users of stimulant substances. Taken together, these findings indicate that substance use should not be used as a reason to withhold PrEP because of concerns about adherence.

We and others have shown that substance use in general, and methamphetamine and other stimulant use in particular, is a likely cause of increased sexual risk behavior among MSM and therefore a predictor for HIV acquisition (*3*,*17*–*21*). Thus, HIV-uninfected MSM who use substances should be considered a target population for PrEP. However, substance-using MSM often face major individual barriers (e.g., HIV-related stigma, substance use) and structural barriers (e.g., economic, healthcare) that might reduce linkage and adherence to PrEP (*13*,*22*–*26*).

Adherence is probably the major factor affecting PrEP effectiveness in those linked to PrEP, as outlined by a recently published mathematical model, which showed that increased adherence was the only factor resulting in reductions of the number needed to treat with PrEP to prevent 1 HIV infection (*27*). Our study indicates that substance use was not associated with decreased adherence to PrEP. Notably, in this study, persons who used methamphetamines did not have worse adherence than persons who did not use methamphetamines. A previous qualitative study indicated that barriers to PrEP uptake and adherence differ by type of substance used. In that study, stimulant drug users were more likely to be concerned that substance use would affect PrEP adherence, and were less concerned about HIV stigma as a barrier to PrEP uptake compared with alcohol users (*13*). However, in our study, we did not find an association between stimulant use and PrEP adherence.

Baseline frequent substance use was associated with higher likelihood of study completion, and no associations were found for alcohol use. Some methamphetamine use was associated with lower likelihood of study completion when compared with frequent or no methamphetamine use. Although some methamphetamine use might relate to MSM who use methamphetamines occasionally, in intermittent binges that are more likely to impart risk for loss to care, the reasons for the increased study dropout of these persons remains unknown.

Some or frequent stimulant use at baseline was strongly associated with contracting an incident STI during the study. This finding was driven mainly by popper use. We also found higher rates of incident STIs for persons with some methamphetamine use when compared with persons who had frequent or no methamphetamine use. One speculative explanation for this association is that many MSM take psychoactive drugs, in particular methamphetamine, and engage in sex at the same time. Also known as chemsex or party and play, this practice is associated with condomless anal sex, multiple sex partners, and the transmission of HIV and other STIs (*4*,*28*,*29*). This intermittent methamphetamine use might not occur frequently (i.e., not fullfilling frequent methamphetamine use in an SCID questionnaire) but might be associated with high-risk sexual activities. Together with the finding that those with some methamphetamine use also have lower study completion rates, this finding might warrant further investigations into tailored HIV prevention counseling, as well as retention counseling, for this group of persons.

As a secondary finding, we found that substantial or severe problems with alcohol use, according to the AUDIT questionnaire at week 48, were associated with lower likelihood of adequate adherence in cross-sectional analysis, although we found no strong association when DBS TFV-DP levels were used as a continuous outcome. Also, we found no strong no associations between baseline AUDIT scores and adherence composites.

Limitations of our study include that DBS TFV-DP levels were only measured at 2 time points, and that the composite adherence outcome logistic regression models did not account for missing follow-up data and time effects. In addition, we assessed frequency of substance use with validated SCID questionnaires that use categories (with the highest category being >5 times) instead of assessing frequency as a continuous outcome. This limitation is applicable particularly to the assessment of alcohol use, in which the frequent use category (i.e., >5 times within 3 months) might not seem appropriate. However, although our study did not look specifically into the effect of more frequent substance and alcohol use (e.g., >10 or >20 times in the previous 3 months), the study included AUDIT and DAST scores that have been accepted as measures of problematic alcohol and substance use.

Another limitation was that IDU was not assessed separately. However, when we analyzed heroin use as a proxy for IDU, we found no negative associations between heroin use and adherence, although these analyses were limited by small sample size. A recent dynamic compartmental cost model suggested oral PrEP for PWIDs a potentially cost-effective strategy to control HIV in regions where IDU is a major driver of the substance use epidemic (*30*). Future studies are needed to evaluate PrEP adherence in PWIDs. Finally, there is a chance that drug interactions could increase TFV-DP concentrations in substance abusers, resulting in misclassification of substance users as adherent, but no evidence supports this hypothesis. In our study, drug interaction seems unlikely, given the consistency of findings across different drugs of abuse, including alcohol, that have different pharmacologic profiles.

In conclusion, for MSM who participated in a randomized controlled trial, we found that baseline substance users had increased STI rates, indicating higher risk behavior, but PrEP adherence was not decreased by substance use. Our findings suggest that substance-using persons are appropriately diligent with PrEP adherence and therefore are excellent candidates for PrEP.

Technical Appendix Additional information on **s**ubstance use and adherence to HIV preexposure prophylaxis for transgender women and men who have sex with men, California, USA.

## References

[1] Freeman P, Walker BC, Harris DR, Garofalo R, Willard N, Ellen JM; Adolescent Trials Network for HIV/AIDS Interventions 016b Team. Methamphetamine use and risk for HIV among young men who have sex with men in 8 US cities. Arch Pediatr Adolesc Med. 2011;165:736–40. 10.1001/archpediatrics.2011.118 21810635 PMC3278965

[2] Santos GM, Coffin PO, Das M, Matheson T, DeMicco E, Raiford JL, et al. Dose-response associations between number and frequency of substance use and high-risk sexual behaviors among HIV-negative substance-using men who have sex with men (SUMSM) in San Francisco. J Acquir Immune Defic Syndr. 2013;63:540–4. 10.1097/QAI.0b013e318293f10b 23572012 PMC4671496

[3] Hoenigl M, Chaillon A, Moore DJ, Morris SR, Smith DM, Little SJ. Clear links between starting methamphetamine and increasing sexual risk behavior: a cohort study among men who have sex with men. J Acquir Immune Defic Syndr. 2016;71:551–7. 10.1097/QAI.0000000000000888 26536321 PMC4788567

[4] Hoenigl M, Chaillon A, Morris SR, Little SJ. HIV infection rates and risk behavior among young men undergoing community-based testing in San Diego. Sci Rep. 2016;6:25927. 10.1038/srep25927 27181715 PMC4867437

[5] Grant RM, Lama JR, Anderson PL, McMahan V, Liu AY, Vargas L, et al.; iPrEx Study Team. Preexposure chemoprophylaxis for HIV prevention in men who have sex with men. N Engl J Med. 2010;363:2587–99. 10.1056/NEJMoa1011205 21091279 PMC3079639

[6] Molina JM, Capitant C, Spire B, Pialoux G, Cotte L, Charreau I, et al.; ANRS IPERGAY Study Group. On-demand preexposure prophylaxis in men at high risk for HIV-1 infection. N Engl J Med. 2015;373:2237–46. 10.1056/NEJMoa1506273 26624850

[7] Baeten JM, Donnell D, Ndase P, Mugo NR, Campbell JD, Wangisi J, et al.; Partners PrEP Study Team. Antiretroviral prophylaxis for HIV prevention in heterosexual men and women. N Engl J Med. 2012;367:399–410. 10.1056/NEJMoa1108524 22784037 PMC3770474

[8] Anderson PL, Glidden DV, Liu A, Buchbinder S, Lama JR, Guanira JV, et al.; iPrEx Study Team. Emtricitabine-tenofovir concentrations and pre-exposure prophylaxis efficacy in men who have sex with men. Sci Transl Med. 2012;4:151ra125. 10.1126/scitranslmed.3004006 22972843 PMC3721979

[9] Choopanya K, Martin M, Suntharasamai P, Sangkum U, Mock PA, Leethochawalit M, et al.; Bangkok Tenofovir Study Group. Antiretroviral prophylaxis for HIV infection in injecting drug users in Bangkok, Thailand (the Bangkok Tenofovir Study): a randomised, double-blind, placebo-controlled phase 3 trial. Lancet. 2013;381:2083–90. 10.1016/S0140-6736(13)61127-7 23769234

[10] Martin M, Vanichseni S, Suntharasamai P, Sangkum U, Mock PA, Chaipung B, et al. Factors associated with the uptake of and adherence to HIV pre-exposure prophylaxis in people who have injected drugs: an observational, open-label extension of the Bangkok Tenofovir Study. Lancet HIV. 2016. 27866873 10.1016/S2352-3018(16)30207-7 PMC11317911

[11] Liu A, Glidden DV, Anderson PL, Amico KR, McMahan V, Mehrotra M, et al.; iPrEx Study team. Patterns and correlates of PrEP drug detection among MSM and transgender women in the Global iPrEx Study. J Acquir Immune Defic Syndr. 2014;67:528–37. 10.1097/QAI.0000000000000351 25230290 PMC4229454

[12] Liu AY, Hessol NA, Vittinghoff E, Amico KR, Kroboth E, Fuchs J, et al. Medication adherence among men who have sex with men at risk for HIV infection in the United States: implications for pre-exposure prophylaxis implementation. AIDS Patient Care STDS. 2014;28:622–7. 10.1089/apc.2014.0195 25396706 PMC4250955

[13] Oldenburg CE, Mitty JA, Biello KB, Closson EF, Safren SA, Mayer KH, et al. Differences in attitudes about HIV pre-exposure prophylaxis use among stimulant versus alcohol using men who have sex with men. AIDS Behav. 2016;20:1451–60. 10.1007/s10461-015-1226-4 26462669 PMC4833721

[14] Moore DJ, Jain S, Dube MP, Daar E, Sun X, Yung J, et al. Randomized controlled trial of daily text messages to support adherence to PrEP in at-risk for HIV individuals: the TAPIR Study. Clin Infect Dis. 2018;66:1566–72. 10.1093/cid/cix1055 29228144 PMC6248545

[15] Castillo-Mancilla JR, Zheng JH, Rower JE, Meditz A, Gardner EM, Predhomme J, et al. Tenofovir, emtricitabine, and tenofovir diphosphate in dried blood spots for determining recent and cumulative drug exposure. AIDS Res Hum Retroviruses. 2013;29:384–90. 10.1089/aid.2012.0089 22935078 PMC3552442

[16] Grant RM, Anderson PL, McMahan V, Liu A, Amico KR, Mehrotra M, et al.; iPrEx study team. Uptake of pre-exposure prophylaxis, sexual practices, and HIV incidence in men and transgender women who have sex with men: a cohort study. Lancet Infect Dis. 2014;14:820–9. 10.1016/S1473-3099(14)70847-3 25065857 PMC6107918

[17] Buchbinder SP, Vittinghoff E, Heagerty PJ, Celum CL, Seage GR III, Judson FN, et al. Sexual risk, nitrite inhalant use, and lack of circumcision associated with HIV seroconversion in men who have sex with men in the United States. J Acquir Immune Defic Syndr. 2005;39:82–9. 10.1097/01.qai.0000134740.41585.f4 15851918

[18] Drumright LN, Little SJ, Strathdee SA, Slymen DJ, Araneta MR, Malcarne VL, et al. Unprotected anal intercourse and substance use among men who have sex with men with recent HIV infection. J Acquir Immune Defic Syndr. 2006;43:344–50. 10.1097/01.qai.0000230530.02212.86 16980913

[19] Koblin BA, Murrill C, Camacho M, Xu G, Liu KL, Raj-Singh S, et al. Amphetamine use and sexual risk among men who have sex with men: results from the National HIV Behavioral Surveillance study—New York City. Subst Use Misuse. 2007;42:1613–28. 10.1080/10826080701212519 17918031

[20] Pines HA, Gorbach PM, Weiss RE, Shoptaw S, Landovitz RJ, Javanbakht M, et al. Sexual risk trajectories among MSM in the United States: implications for pre-exposure prophylaxis delivery. J Acquir Immune Defic Syndr. 2014;65:579–86. 10.1097/QAI.0000000000000101 24378726 PMC4026016

[21] Hoenigl M, Anderson CM, Green N, Mehta SR, Smith DM, Little SJ. Repeat HIV-testing is associated with an increase in behavioral risk among men who have sex with men: a cohort study. BMC Med. 2015;13:218. 10.1186/s12916-015-0458-5 26444673 PMC4596465

[22] Mimiaga MJ, Closson EF, Kothary V, Mitty JA. Sexual partnerships and considerations for HIV antiretroviral pre-exposure prophylaxis utilization among high-risk substance using men who have sex with men. Arch Sex Behav. 2014;43:99–106. 10.1007/s10508-013-0208-8 24243002 PMC4532732

[23] Escudero DJ, Kerr T, Wood E, Nguyen P, Lurie MN, Sued O, et al. Acceptability of HIV pre-exposure prophylaxis (PREP) among people who inject drugs (PWID) in a Canadian setting. AIDS Behav. 2015;19:752–7. 10.1007/s10461-014-0867-z 25086669 PMC4315758

[24] Guise A, Albers ER, Strathdee SA. ‘PrEP is not ready for our community, and our community is not ready for PrEP’: pre-exposure prophylaxis for HIV for people who inject drugs and limits to the HIV prevention response. Addiction. 2017;112:572–8. 10.1111/add.13437 27273843 PMC5145792

[25] Edelman EJ, Moore BA, Calabrese SK, Berkenblit G, Cunningham C, Patel V, et al. Primary care physicians’ willingness to prescribe HIV pre-exposure prophylaxis for people who inject drugs. AIDS Behav. 2017;21:1025–33. 10.1007/s10461-016-1612-6 27896552 PMC5344709

[26] Marshall BD, Milloy MJ. Improving the effectiveness and delivery of pre-exposure prophylaxis (PrEP) to people who inject drugs. Addiction. 2016. 27730702 10.1111/add.13597 PMC6659115

[27] Jenness SM, Goodreau SM, Rosenberg E, Beylerian EN, Hoover KW, Smith DK, et al. Impact of the Centers for Disease Control’s HIV preexposure prophylaxis guidelines for men who have sex with men in the United States. J Infect Dis. 2016;214:1800–7. 10.1093/infdis/jiw223 27418048 PMC5142082

[28] Grov C, Rendina HJ, Breslow AS, Ventuneac A, Adelson S, Parsons JT. Characteristics of men who have sex with men (MSM) who attend sex parties: results from a national online sample in the USA. Sex Transm Infect. 2014;90:26–32. 10.1136/sextrans-2013-051094 24052337 PMC3927726

[29] Hegazi A, Lee MJ, Whittaker W, Green S, Simms R, Cutts R, et al. Chemsex and the city: sexualised substance use in gay bisexual and other men who have sex with men attending sexual health clinics. Int J STD AIDS. 2017;28:362–6. 10.1177/0956462416651229 27178067

[30] AIDSVu. Rollins School of Public Health, Emory University [cited 2017 Dec 2]. https://aidsvu.org

