# Supplementary material for: Substance Use and Adherence to HIV Preexposure Prophylaxis for Men Who Have Sex with Men
Source: Emerg Infect Dis. 2018 Dec;24(12):2292–302. doi: 10.3201/eid2412.180400 (PMC6256399; doi:10.3201/eid2412.180400)
Supplement: Technical Appendix — Additional information on substance use and adherence to HIV preexposure prophylaxis for transgender women and men who have sex with men, California, USA. [file 18-0400-Techapp-s1.pdf]

Article DOI: https://doi.org/10.3201/eid2412.18-0400

Substance Use and Adherence to HIV Preexposure Prophylaxis for Men who Have Sex with Men

Technical Appendix

Technical Appendix Table 1. Associations of substance use at weeks 12 and 48 with adequate (DBS TFV-DP level >719 fmoL/punch) and near-perfect (DBS TFV-DP level >1,246 fmoL/punch) MSM*

Substance use at wk 12, n = 357	DBS TFV-DP level ≤719 fmoL/punch	DBS TFV-DP level >719 fmoL/punch	p value	DBS TFV-DP level ≤1,246 fmoL/punch	DBS TFV-DP level >1,246 fmoL/punch	p value
Methamphetamine			0.57			0.06
No	33 (11)	266 (89)		157 (53)	142 (47)	
Some	5 (14)	31 (86)		19 (53)	17 (47)	
Frequent	1 (5)	21 (95)		10 (45)	12 (55)	
Heroin			0.73			0.57
No	38 (11)	308 (89)		812 (53)	164 (47)	
Some	1 (17)	5 (83)		2 (33)	4 (67)	
Frequent	0 (0)	5 (100)		2 (40)	3 (60)	
Marijuana			0.06			0.39
No	20 (9)	193 (91)		105 (49)	108 (51)	
Some	13 (19)	54 (81)		39 (58)	28 (42)	
Frequent	6 (8)	71 (92)		42 (55)	35 (45)	
Cocaine			0.69			0.94
No	32 (11)	270 (89)		157 (52)	145 (48)	
Some	5 (12)	38 (88)		22 (51)	21 (49)	
Frequent	2 (17)	10 (83)		7 (58)	5 (42)	
Poppers (nitrite)			0.19			0.41
No	24 (13)	168 (88)		100 (52)	92 (48)	
Some	10 (13)	68 (87)		45 (58)	33 (42)	
Frequent	5 (6)	82 (94)		41 (47)	46 (53)	
Alcohol			0.39			0.55
No	9 (15)	50 (85)		27 (46)	32 (54)	
Some	13 (11)	101 (89)		60 (53)	54 (47)	
Frequent	17 (9)	167 (91)		99 (54)	85 (46)	
Any substance†			0.26			0.42
No	11 (10)	95 (90)		53 (50)	53 (50)	
Some	16 (15)	90 (85)		61 (58)	45 (42)	
Frequent	12 (8)	133 (92)		72 (50)	73 (50)	
Substance use at wk 48, n = 311‡						
Methamphetamine			0.95			0.73
No	46 (17)	221 (83)		151 (57)	116 (43)	
Some	4 (19)	17 (81)		12 (57)	9 (43)	
Frequent	4 (17)	19 (83)		11 (48)	12 (52)	
Marijuana			0.07			0.61
No	30 (17)	146 (83)		95 (54)	81 (46)	
Some	16 (26)	45 (74)		34 (56)	27 (44)	
Frequent	8 (11)	66 (89)		45 (61)	29 (39)	
Poppers (nitrite)			0.52			0.48
No	31 (20)	125 (80)		91 (58)	65 (42)	
Some	11 (14)	68 (86)		45 (57)	34 (43)	
Frequent	12 (16)	64 (84)		38 (50)	38 (50)	
Alcohol			0.64			0.61
No	11 (21)	42 (79)		33 (62)	20 (38)	
Some	17 (18)	75 (82)		50 (54)	42 (46)	
Frequent	26 (16)	140 (84)		91 (55)	75 (45)	
Any substance†			0.45			0.69
No	19 (22)	69 (78)		47 (53)	41 (47)	
Some	15 916)	79 (84)		56 (60)	38 (40)	

Substance use at wk 12, n = 357	DBS TFV-DP level ≤719 fmoL/punch	DBS TFV-DP level >719 fmoL/punch	p value	DBS TFV-DP level ≤1,246 fmoL/punch	DBS TFV-DP level >1,246 fmoL/punch	p value
Frequent	10 (16)	109 (84)		71 (55)	58 (45)	
AUDIT category at wk 48			**0.03**			0.83
<8	45 (18)	204 (82)		140 (56)	109 (44)	
8–15	5 (10)	47 (90)		28 (54)	24 (46)	
>15	4 (44)	5 (56)		6 (67)	3 (33)	
DAST10 problems category at wk 48			0.10			**0.04**
No/low	41 (18)	186 (82)		128 (57)	99 (44)	
Moderate	13 (20)	52 (80)		41 (63)	24 (37)	
Substantial/severe	0 (0)	18 (100)		5 928)	13 (72)	

*Values are no. (%). Bold indicate statistical significance. AUDIT, Alcohol Use Disorders Identification Test; DAST, Drug Abuse Screening Test; DBS, dried blood spot; MSM, men who have sex with men; TSF-DV, tenofovir diphosphate.
†Marijuana and alcohol excluded.
‡Data for heroin and cocaine not shown; for both, all p values >0.5.

Technical Appendix Table 2. Multivariable logistic regression models to assess the association of baseline substance use and stimulant use at study completion and incident STIs for MSM*

Variables for predicting study completion	OR (95% CI)	p value
Model 1†		
Intervention arm (receiving individualized texting for adherence to daily TDF/FTC)	0.683 (0.405–1.149)	0.1507
Baseline some substance use (any)	1.398 (0.746–2.617)	0.2959
Baseline frequent substance use (any)	1.927 (1.014–3.661)	0.0452
Variables for predicting incident STI during study		
Model 2†		
Intervention arm	0.906 (0.589–1.394)	0.654
Age	0.965 (0.941–0.989)	0.004
Baseline some stimulant use	3.352 (1.945–5.777)	<0.001
Baseline frequent stimulant use	3.496 (2.013–6.072)	<0.001
Positive STI test result at baseline	1.601 (0.992–2.585)	0.054

*FTC, emtricitibine; MSM, men who have sex with men; OR, odds ratio; STI, sexually transmitted infection; TDF, tenofovir disoproxil fumarate.
†Hosmer and Lemeshow goodness of fit test for model 1: χ^2 = 0.375 (df = 2); p = 0.829. Hosmer and Lemeshow goodness of fit test for model 2: χ^2 = 2.892 (df = 8); p = 0.941.
